# Supplementary material for: Isothermal anisotropic magnetoresistance in antiferromagnetic metallic IrMn
Source: Sci Rep. 2016 Oct 20;6:35471. doi: 10.1038/srep35471 (PMC5071853; doi:10.1038/srep35471)
Supplement: Supplementary Information [file srep35471-s1.pdf]

Supplementary Information on: Isothermal anisotropic magnetoresistance in antiferromagnetic metallic IrMn

R. Galceran, I.Fina*, J. Cisneros-Fernández, B. Bozzo, C. Frontera, L. López-Mir, H. Deniz, K.-W. Park, B.-G. Park, Ll. Balcells, X.Martí, T. Jungwirth, B. Martínez

Figure S1. Detailed structural characterization of LCMO film

Figure S1(a) shows the reciprocal space map of (103) diffraction peak of a STO/LCMO film. This shows the high epitaxial character of the growth and that film is fully strained. The good quality of the film can be also observed in the zoom of the STEM cross-section image of the LCMO film shown by figure S1(b).

Synchrotron X-ray diffraction of figure S1a has been done at KMC2 beamline (BESSY-II at Helmholtz-Zentrum, Berlin), which is equipped with a two dimensional General Area Detector Diffraction System.

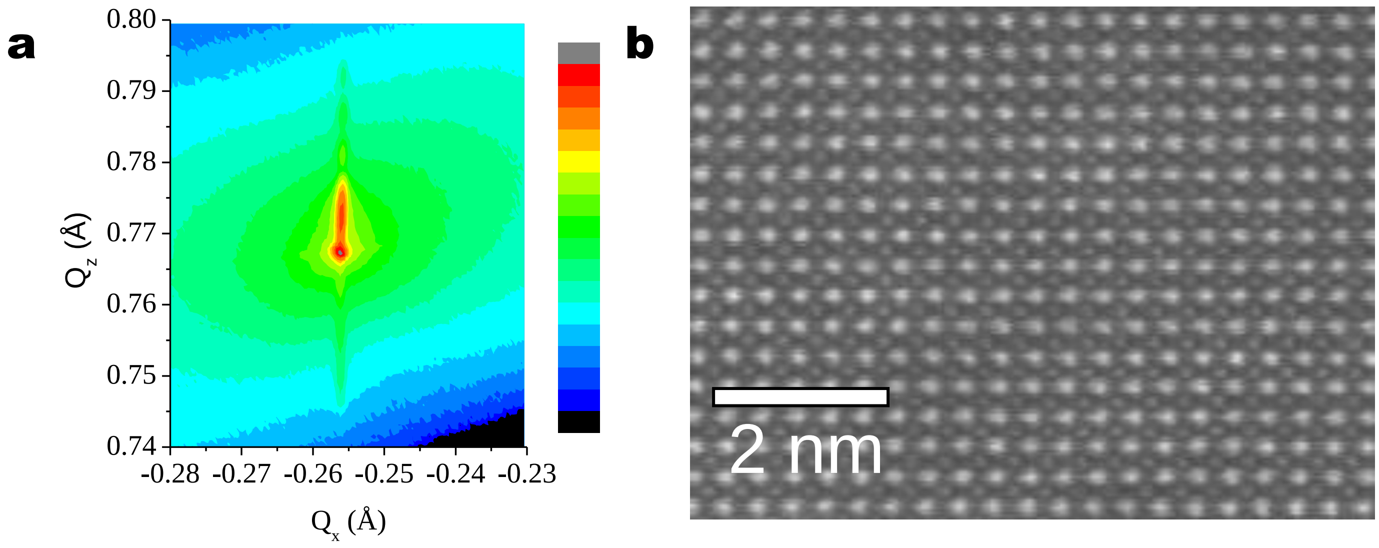

*ignasifinamartinez@gmail.com

Figure S2. Transmission electron microscopy image of the IrMn layer

The zoom of the TEM cross-section of antiferromagnetic-metallic (IrMn, 2.2 nm)/ferromagnetic-insulator (La2CoMnO6, LCMO) bilayer, shown in figure S2, reveals that the IrMn layer has a grainy morphology. Several grains are crystalline (signalled by arrows). Other grains show no- clear crystallinity, because of the non-proper orientation of the crystalline planes with respect to electron beam, signalling the polycrystalline nature of IrMn layer.

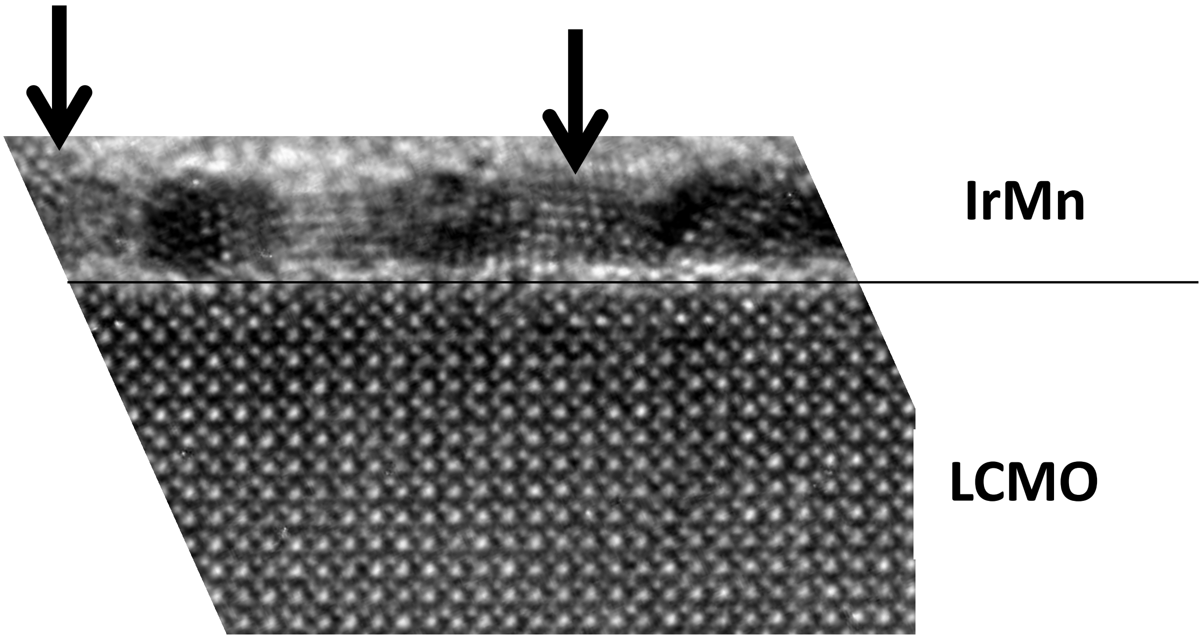

Figure S3. Anisotropic magnetoresistance of IrMn(10 nm)/LCMO bilayer

In figure S3, we show the AMR = (R(Φ)-R(Φ=0))/R(Φ=0) measurements performed at 5 K under a magnetic field of 3 T for a IrMn(10 nm)/LCMO film (after field-cooling the samples at 2 T Φ=0º).. For comparison, we have also included the result for the IrMn(2.2 nm)/LCMO sample of the main manuscript (after field-cooling the samples at 2 T Φ=0º and Φ=90º). It can be observed that no-sizeable signal is observed for the 10 nm IrMn film.

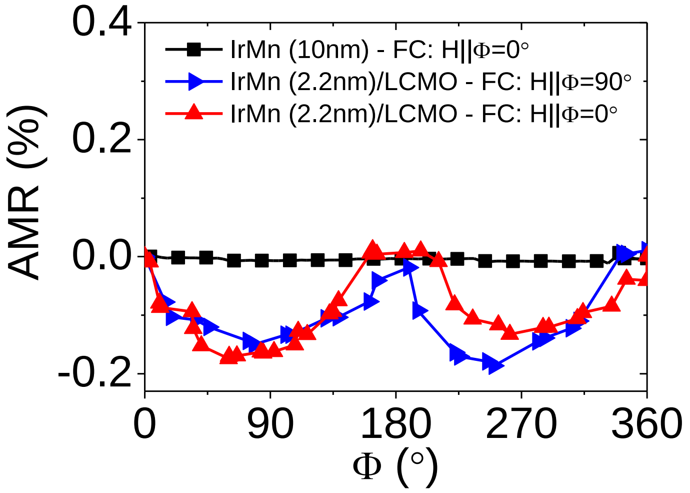

Table S1. Fitting results for the exchange bias

Table S1 shows the values of the fits obtained by the expression $M_0 \cdot \tanh[(H-H_C)/\delta H] + \chi_{PM}$ after field cooling the sample with magnetic field applied along opposite directions as indicated in the first raw.

	Field cooling:	Negative 1	Positive 1	Negative 2	Positive 2
		Value	Value	Value	Value
Increasing H branch	M_0 (emu·10^{-5})	7.2	6.7	7.2	6.3
	H_c (mT)	623	618	640	627
	δH (mT)	1890	1739	1886	1645
	χ (emu·10^{-13}/T)	3.56	3.44	3.54	3.40
Decreasing H branch	M_0 (emu·10^{-5})	6.3	7.1	6.4	6.4
	H_c (mT)	-620	-622	-638	-630
	δH (mT)	1717	1883	1677	1677
	χ (emu·10^{-13}/T)	3.36	3.49	3.38	3.38
	H_{EB} (mT)	1.5	-2	1	-1.5
